# The Taunton and Somerset Hospital

**Published:** 1890-03-08

**Authors:** 


### The Hospital Workshop.

### No. XXII.—THE TAUNTON AND SOMERSET HOSPITAL.

(By our Special Commissioner.)

This charity has been in existence for the last eighty years, during which time it has carried on important work in the county of Somerset. It lies on the outskirts of the town, facing the street that winds its broad length from one end of Taunton to the other. Its appearance is somewhat striking. The main entrance opens on to the first floor, and is reached by a portico with a double flight of steps, an arrangement which, though undoubtedly effective from an architectural point of view, must add considerably to the task of carrying in helpless patients. The building is plain and substantial, and sanitary appliances are modern. In the library hangs a surveyor's plan of all drains, with particulars appended. The advantage of a means of ready reference in matters of this kind is obvious, and if some such method were more generally adopted, committees would be saved much unnecessary trouble and expense. Within the last year or two numerous alterations have taken place in the arrangements of the hospital. Among others, may be mentioned the establishment of a casualty ward, provision of fire appliances, and the reorganisation of the nursing staff. Many of these improvements are due to the energy of the House Surgeon (Mr. W. Burrough Cosens), who, for instance, personally raised the subscriptions needed for providing eight cots for the use of the children.

The wards are large, lofty, and comfortable, and can accommodate rather more than a hundred patients. Heating is effected by means of open fire places and hot-air pipes, and ventilation by the windows, and by numerous air valves and gratings at various levels. The floors are of plain deal. The furniture displays a curious blending of the old style and the new. In the centre of the floor, for instance, stands a spick-and-span surgeon's table, running on wheels with noiseless rubber tires. Its top is covered with white tiles, at the sides are tin-lined shelves for dressings, while below is a roomy drawer for bandages and other requisites. Now let us turn to the beds. The framework of the bedstead is of iron, fitted with a panelled boarding at the head. A shelf rests on top of this wooden structure, which is painted white, and invariably bears a distinct impression of the heads of past and present patients. The bed itself is made of flock, resting on steel laths, and anything more uncomfortable or faulty from a sanitary point of view it would be hard to imagine. Many of the patients, it is pleasant to note, have not lost their healthy country colour. Here is a girl of twenty who has kept her cherry cheeks, in spite of the fact that she has been ten weeks in the hospital suffering from "psoas" abscess. The usual cause of that malady is disease of one of the spinal bones, and the abscess burrows along the sheath of a muscle that stretches from backbone to thigh. These cases were formerly very chronic and fatal, but antiseptic methods have made a wonderful improvement in results. In this instance, on December 8th, 23 ounces of pus were drawn off by the aspirator, 12 ounces on the 11th, 20 on the 17th, 14 on the 30th. On January 16th half an ounce only was taken away; 2 drachms on the 26th; and half a drachm on February 10th. It should be noted, moreover, that on the last four occasions the matter was quite thin. The patient will almost certainly make a speedy and perfect recovery.

Results, as a rule, are good. This is owing in great measure no doubt, to favourable surroundings, and also to the fact that many of the patients are healthy country people. Anaemia is common; a fact which is sometimes attributed to the relaxing nature of the climate. It is also more or less traceable to the influence of the factories which are scattered broadcast throughout the country. Accidents are common, many being connected with the pursuits and occupations of rural life. Here is a ruddy-faced labourer who has severed the tendons at the back of his left arm by a blow with a bill-hook whilst "hedging." The cut tendons have been carefully stitched together and the wound dressed antiseptically. Near by is a farmer whose knee-cap has been fractured by the kick of a horse. In the medical wards are a couple of cases of pneumonia following influenza. One villager is suffering from extensive lead paralysis, caused by drinking cider. There seems to be a large number of heart affections, which may be partly explained by the prevalence of rheumatism in the low-lying and water-laden plains that make up so large a portion of Somerset.

The building of the Victoria Jubilee Nursing Institute—an important feature of the charity—adjoins the hospital, with which it is in communication by corridors fitted with fireproof doors. There is accommodation for about thirty nurses, fourteen of whom are engaged in the wards, while the rest form a staff of skilled attendants for private work in the neighbourhood. The place is under the care of a "Lady Superintendent of Nurses." The additional post of "Matron" is at first a little puzzling, until one learns that the last-named has no duties whatever beyond housekeeping. The home is bright and cheerful, and provided with excellent rooms for various purposes. Among the latter is a nurses' class-room, where a course of lectures is given by the House Surgeon on elementary anatomy and physiology, and on nursing by the Lady Superintendent. The basement floor makes a handsome hall for out-patients, and its arrangements, including a coffee-stall, seem very nearly, if not quite, perfect.

Although much good has been effected by the House Surgeon during his two years of residence, there are still some details that might be altered with advantage. For instance, the present bath for the men is awkwardly placed and badly fitted. Again, most people, on entering so imposing an edifice, would expect to find the porter in livery; but there is nothing in his dress to distinguish that official from a casual visitor or a patient. Then there are the convalescent day-rooms, most excellent in their way, but quite spoiled by the bare and depressing way in which they are furnished. Enough has been said about the beds and bedding, the sooner they are done away with the better. These suggestions, be it understood, are offered in a friendly spirit, and with a full recognition of the zeal and earnestness of the committee, who are hindered by lack of funds from carrying out reforms which they are quite willing to admit as necessary. A further point worthy of consideration is that the House Surgeon appears to have more work than should fall to the lot of one man to perform. He acts as registrar, dresses all important surgical cases, attends out-patients three times a week, treats all casualties, serious or trifling; performs *post mortem* examinations, takes care of library, lectures to nurses; and, in addition to a multitude of other duties, acts as secretary to the Jubilee Institute. Nothing could be easier than to obtain a qualified man to fill the post of junior House Surgeon; and in these days of keen competition there would be no difficulty in getting such services in return for board and lodging. That kind of parsimony, however, cannot be recommended to any solvent institution, which of necessity already derives most essential aid from the gratuitous services of members of the medical profession. In a county like Somerset there should be no permanent want of funds to meet the "growing wants and increasing expenses" of this most excellent charity, concerning which the words of the annual report may be heartily endorsed, when it says that "its usefulness can hardly be over-estimated."

